# Diverse roles of YTHDC1 in chromatin and blood cancers

**DOI:** 10.3389/fgene.2026.1773468

**Published:** 2026-05-08

**Authors:** Baptiste Pernon, Yacine Benchikh, Cyril Fournier, Maxime Delforge, Baptiste Dumétier, Laurent Delva, Amandine Durand, Cédric Rossi, Laurent Martin, Fabien Guidez, André Verdel, Romain Aucagne, Mary B. Callanan

**Affiliations:** 1 Université Bourgogne Europe, Inserm, CTM UMR 1231, Dijon, France; 2 Laboratory for Innovation in Genetics and Epigenetics in Oncology (IGEO), CHU Dijon Bourgogne, Dijon, France; 3 Department of Clinical Hematology, CHU Dijon Bourgogne, Dijon, France; 4 Department of Pathology, CHU Dijon Bourgogne, Dijon, France; 5 Université Grenoble Alpes, Inserm U1209, CNRS UMR 5309, Institute for Advanced Biosciences, La Tronche, France

**Keywords:** class switch recombination, condensates, enhancers, epitranscriptome, m6A inhibitors, MYC, non-coding RNA, p53

## Abstract

Epitranscriptomics, the study of RNA modifications, together with their functional characterization, is emerging as an important area of investigation in RNA biology. Of the over 170 RNA modifications that have been identified on mRNA and non-coding RNAs, N6-methyladenosine (m^6^A) modification to mRNA is recognized as a key regulator of gene expression, splicing and protein translation. Functional readout of m^6^A is mediated by m^6^A readers mostly in the cytoplasm except for the nuclear-localized YTHDC1. m^6^A-YTHDC1 function has recently been extended to include short and long-range fine-tuning of genome activity via chromatin-associated mechanisms. This review summarizes YTHDC1-m^6^A nuclear functions in normal and cancer cells with special focus on its chromatin-associated roles and the ability of YTHDC1 to assemble into higher order nuclear structures called condensates. These processes are disturbed in cancer.

## 1 Introduction

**FIGURE 1:**
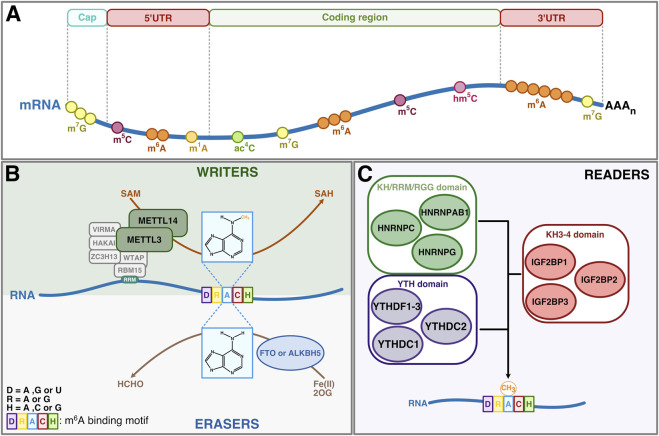
m^6^A regulation by writers, erasers and readers. **(A)** Numerous chemical modifications occur in RNA and localize to distinct regions of mRNA or other RNA substrates. In mRNA, m^6^A is mainly enriched in the 3′UTR region. Other modifications, such as m^1^A, ac^4^C, m^5^C and hm^5^C, are found in the 5′UTR and coding sequence. **(B)** The N^6^-adenosine (m^6^A) modification is catalyzed by writer enzymes, primarily the m^6^A-METTL complex, which includes the methyltransferase METTL3, its non-catalytic partner METTL14, the adaptor subunit WTAP and associated components comprising VIRMA, HAKAI, ZC3H13 and RBM15 (see text for details). m^6^A can be removed by the RNA demethylases FTO or ALKBH5. **(C)** The m^6^A modification is recognized by different families of RNA-binding proteins (RBPs), with the YTH domain protein family being the best characterized. Other RBPs, including IGF2BP1-3 and members of the HNRNP family (nuclear), can also bind m^6^A. KH: K Homology; RRM: RNA recognition motif; RGG: Arginine/Glycine Rich domain.

Chemical modifications of RNA have been identified on mRNA and various non-coding RNAs and these can be broadly divided, at least for mRNA, into cap modifications and internal modifications. Of the latter, the most abundant internal modification in eukaryotic cells is N6-methyladenosine (m^6^A) (Meyer et al., 2012; Dominissini et al., 2013). Considerable progress has been made on understanding the dynamics of m^6^A deposition and function and, in a parallel with epigenetics, RNA m^6^A methyltransferases are referred to as ‘writers’, m^6^A-binding proteins as ‘readers’ and the RNA demethylases as ‘erasers’ (Figure 1).

Among the m^6^A readers are the YTH family of RNA binding proteins (RBP) of which YTHDC1 is the sole member to be exclusively nuclear. First described as a regulator of RNA splicing (Xiao et al., 2016), YTHDC1 is emerging as a crucial regulator of transcription and gene expression, endogenous retroviral element silencing, and genome integrity, through its activity in m^6^A-dependent chromatin remodeling and nuclear organization (Widagdo et al., 2022). By multiple mechanisms operating at chromatin, m^6^A-YTHDC1 has now been found to play critical roles in diverse biological processes including the self-renewal and differentiation of embryonic (Liu et al., 2021) and hematopoietic stem cells (Sheng et al., 2021; Zuo et al., 2024), cell stress (Timcheva et al., 2022), class switch recombination (Nair et al., 2021) or DNA damage response and repair pathways (Elvira-Blazquez et al., 2024; Tsao et al., 2025).

## 2 Writing, erasing and reading the m^6^A methylome

### 2.1 m^6^A writers

m^6^A occurs co-transcriptionally at a consensus sequence, DRACH (D = A, G or U, R = A or G; H = A, C or U), with the central A being the substrate for methylation, primarily within the last exon, near the stop codon, and at the 3′UTR region. The DRACH consensus sequence appears to be conserved between human and mouse (Meyer et al., 2012; Dominissini et al., 2013; Tegowski et al., 2022). The methyl donor for m^6^A methylation is S-adenosylmethionine (SAM). By single cell DART-seq, it has been found that m^6^A is highly heterogeneous at the single-cell level with most m^6^A sites occurring in a small proportion of cells and significant differential methylation across subpopulations of cells (Tegowski et al., 2022).

Biochemical and structural biology investigations have identified METTL3, a member of the seven-beta-strand (7BS) methyltransferase-like (METTL) family of protein and RNA methyltransferases, as the major m^6^A methylase for mRNA (Falnes, 2024; Hobartner et al., 2024). METTL3 operates within a complex that is composed of a core catalytic subunit comprised of a heterodimer of METTL3, which contains the m^6^A methylase activity, and METTL14, a non-catalytic partner (Hobartner et al., 2024). Accessory regulatory proteins that assist and/or stabilize RNA binding on various substrates to the core complex have been described. These include Wilms’ tumor 1-associated protein (WTAP), KIAA1429 (VIRMA), Zinc finger CCCH domain-containing protein 13 (ZC3H13), RNA binding motif protein 15/15 paralog (RBM15/15B), and E3 ubiquitin ligase CBLL1 (HAKAI) (Hobartner et al., 2024). Regarding the interactions between methylation complex subunits, METTL14 binds exclusively with METTL3 through a methyltransferase domain interaction, while METTL3 interacts with the adaptor protein WTAP. WTAP, in turn interacts with VIRMA, HAKAI and ZC3H13 (Hobartner et al., 2024).

The co-transcriptional m^6^A marking activity of the METTL3-METTL14 RNA methyltransferase complex has been proposed to involve recruitment to trimethylated histone H3 lysine 36 (H3K36me3), a chromatin mark of active transcription, and interaction with RNA polymerase (Huang et al., 2019).

Several biochemical and structural studies have provided mechanistic insights into METTL3-METTL14 methylase activity (Oerum et al., 2021). Most recently, by using crystallographic and substrate-mimic approaches, a two-step mechanism of action has been proposed. A novel conserved pocket, essential for m^6^A recognition, has been identified adjacent to the SAM binding domain of the METTL3-METTL14 complex, and this pocket swivels the target A upon its conversion to m^6^A thus switching METTL3-METTL14 from an m^6^A writer to sensor (Qi et al., 2024).

Other enzymes in the METTL family of RNA methyltransferases are METTL16 and METTL5, and these have distinct methylation sites and activities, different to those mediated by METTL4. For example, METTL16 methylates U6 small nuclear non-coding RNA (snRNA), a process required for efficient and accurate splicing of specific pre-mRNAs, and mRNA encoding the enzyme SAM synthetase MAT2A (methionine adenosyltransferase 2A), at sites within the 3′UTR (Pendleton et al., 2017; Shima et al., 2017). Specifically, in high SAM conditions, METTL16-mediated methylation of the MAT2A transcript occurs at conserved hairpins in the 3′UTR region, resulting in its nuclear retention. In low SAM conditions, the latter is reversed, resulting in nuclear export and translation of the SAM synthetase, thus establishing a novel regulatory loop for control of cellular SAM by METTL16 activity (Pendleton et al., 2017; Shima et al., 2017). This process has been shown to critically depend on the m^6^A reader, YTHDC1, which upon binding the m^6^A-modified *MAT2A* 3′UTR, appears to promote transcript degradation (Shima et al., 2017). The motif that is targeted by METTL16 methylation activity within the conserved hairpins is UACA*GAR, where A* denotes the methylation site. Aspects of METTL16-dependent regulation of *MAT2A* appear to operate in a cell type-specific manner. For example, in murine B cells and HeLa cells, *MAT2A* splicing does not appear to play a role (Shima et al., 2017). By contrast, in HEK293 cells, METTL16 seems to control *MAT2A* expression and activity, in SAM-replete versus SAM-depleted conditions, through a dual mechanism involving splicing control (intron retention) and 3′UTR methylation in *MAT2A* pre-mRNA (Pendleton et al., 2017). Taken together, abrogation of METTL16-mediated regulation of *MAT2A* has widespread consequences for SAM-dependent cellular processes, supporting the notion that METTL16 is a critical contributor to cellular maintenance of SAM homeostasis (Flaherty et al., 2025).

METTL5 and ZCCHC4 are mainly involved in ribosome subunit methylation and are enriched in the nucleoli and nucleus, corresponding to the sites of ribosome biogenesis (Shi et al., 2019). Briefly, it has been demonstrated that m^6^A modification at sites 1832, in 18S rRNA, and 4220 in 28S rRNA, is mediated by METTL5 and ZCCHC4, respectively (Ma et al., 2019; van Tran et al., 2019).

RNA methyltransferases generating m^6^A thus display distinct RNA substrate and motif specificities that are likely to underpin diverse functional roles in physiology and pathology, particularly cancer. Detailed mechanistic studies will be needed to uncover the cell and cancer type context-dependent activities that offer potential for uncovered novel disease mechanisms and therapeutic strategies (see these recent reviews for expert mechanistic and methodological considerations and perspectives: Deng et al., 2026; Luo et al., 2026).

### 2.2 m^6^A erasers

The m6A modification is reversible through the action of RNA demethylases (Shi et al., 2019). Two main RNA demethylases are characterized to date: FTO (Fat mass and obesity-associated protein) and ALKBH5 (AlkB homolog 5) (reviewed in detail elsewhere), respectively. Both FTO and ALKBH5 belong to the nonheme Fe(II)-2-oxoglutarate (2OG)-dependent dioxygenase AlkB family proteins that repair N-alkylated nucleobases by oxidative demethylation in DNA and RNA. Deregulated RNA demethylase activity is manifest in solid and blood cancers thus pointing to tight control of this activity in normal tissues (Barbieri and Kouzarides, 2020).

### 2.3 m^6^A readers

**FIGURE 2:**
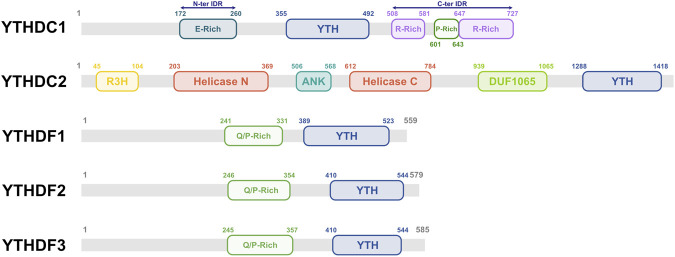
Human YTH domain family of m^6^A RNA binding proteins. Schematic representation of the five members of the YTH domain protein family, as indicated. The amino acid residues involved in m^6^A binding are indicated within the YTH domain of each protein (see text for details).

Among the best-characterized m^6^A reader proteins is the YT521-B homology (YTH) domain protein family, comprising five members: YTHDF1, YTHDF2, YTHDF3, YTHDC1, and YTHDC2 (Patil et al., 2018). These proteins share an evolutionarily conserved 100–150 amino acid domain (YT521-B homology domain) which contains tryptophan residues (W377 and W428 in human YTHDC1) that form an aromatic cage and binding pocket for m^6^A within the consensus DRACH motif (Figures 1, 2, respectively) (Widagdo et al., 2022).

Binding of m^6^A-modified RNA is also mediated by other proteins that recognize m^6^A through distinct domains. For instance, IGF2BP1-3 (insulin-like growth factor 2 mRNA binding protein) interacts with m^6^A via a KH3-4 domain. RNA binding by the heterogenous nuclear ribonucleoproteins family (HNRNPC, HNRNPG and HNRNPA2B1) is more complex and occurs through KH, RRM and RGG domains, respectively, through an ‘m^6^A switch’ mechanism whereby m^6^A promotes RNA unfolding and increases accessibility to nearby binding sites (Liu et al., 2015; Porman et al., 2022). Additionally, PRRC2A (proline-rich and coiled-coil-containing protein 2A), has been identified as an m^6^A binding protein, predominantly in testes, although the specific RNA-binding domain remains to be identified (Tan et al., 2023). The RNA binding activities, m^6^A switch mechanisms and biological functions of these proteins are reviewed elsewhere (Figure 1) (Shi et al., 2019; Barbieri and Kouzarides, 2020).

## 3 YTH family of m^6^A RNA readers

Among the YTH domain family of m^6^A readers, YTHDF1, YTHDF2, and YTHDF3 are primarily localized in the cytoplasm. YTH domain-containing 2 (YTHDC2) is localized to the nucleus and the cytoplasm whereas YTHDC1 is the sole member to be exclusively nuclear (Widagdo et al., 2022). YTHDF1 promotes m^6^A-modified RNA translation by enhancing its efficiency. YTHDF2 is involved in m^6^A transcript stability regulation through RNA decay. YTHDF3 plays a role in both functions. The YTH domain-containing two protein (YTHDC2) has been implicated in m^6^A RNA stability, RNA degradation and meiosis. This protein appears to function as an RNA-induced ATPase and has 3′–5′ RNA helicase activity, which may be involved in the reorganization of protein structures (Widagdo et al., 2022).

YTHDC1 functions are numerous and include regulation of chromatin-dependent processes such as X chromosome inactivation, gene and retroelement silencing, transcription-coupled RNA splicing, enhancer activity via enhancer RNA (eRNA) and organization of chromatin topology in the nucleus, in particular through its ability to form molecular condensates. More recently, YTHDC1 has been shown to play a role in immunoglobulin gene class switch recombination (Nair et al., 2021) and in DNA damage response pathways (Elvira-Blazquez et al., 2024; Tsao et al., 2025). Abundant literature now points to YTHDC1 as a target for misregulation in cancer where, depending on cell context, oncogene or tumor suppressor functions are suspected thus pointing to tight control of YTHDC1 expression and function in normal somatic cells (Barbieri and Kouzarides, 2020; Deng et al., 2026; Luo et al., 2026). Also, YTHDC1 depletion (but not other YTH members) leads to early embryonic lethality in mice (Kasowitz et al., 2018).

### 3.1 YTHDC1 structure and function

The human YTHDC1 protein (84.7 kDa) is composed of 727 amino acids and contains a highly conserved YTH domain (also known as the YT521-B homology domain) localized between amino acid positions 355 and 492 (Figure 2). Binding by YTHDC1 of m^6^A in RNA occurs as a monomer and involves three conserved amino acids: W377, W428, and L439. Crystallographic analyses have revealed that this interaction is stabilized by four hydrogen bonds involving N363, N367, and S378. The m^6^A motif binding specificity of YTHDC1 is similar to that seen for other YTH protein family members except that YTHDC1 appears to show preferential binding to the GG (m^6^A)C motif (Xu et al., 2014; Xu et al., 2015). The specificity of YTHDC1 for the GGAC motif can be explained by the formation of an additional hydrogen bond between the n-1 guanosine of the motif and V382 of YTHDC1. Replacing G–1 with any other nucleotide would disrupt this hydrogen bond. Furthermore, replacing G–1 with adenosine could introduce steric clashes with V382, between the NH2 group of adenosine and the main chain NH group of V382. Motif specificity is reinforced by a further two hydrogen bonds between the n-2 guanosine and D476 of the YTH domain of YTHDC1 (Xu et al., 2014; Xu et al., 2015).

### 3.2 YTHDC1 and intrinsically disordered regions

The YTH domain of human YTHDC1 is flanked by two intrinsically disordered regions (IDRs), an N-terminal glutamate-rich IDR1 and a C-terminal arginine/proline rich IDR2 (Figure 2) (Liu et al., 2020; Cheng et al., 2021). IDR-containing proteins have a high tendency to undergo liquid-liquid phase separation (LLPS) to form membrane-less macromolecular structures called condensates. YTHDC1-m^6^A interactions promote nuclear condensation and assembly of condensates which are essential for the activity of certain regulatory non-coding RNA functions, notably enhancer RNAs (eRNAs) (Liu et al., 2020). Importantly, YTHDC1 condensate activity is a target for misregulation in leukemia (Cheng et al., 2021).

### 3.3 YTHDC1 and post-translational modifications

Post-translational modifications of YTHDC1 have been described and these can regulate YTHDC1 function via control of YTHDC1 stability and/or distribution in the nucleus.

YTHDC1 (YT521-B) has been shown to be tyrosine phosphorylated and this controls its distribution in the nucleoplasm (Rafalska et al., 2004). Specifically, tyrosine phosphorylation appears to cause sequestration of YTHDC1 in an insoluble nuclear form, which at least in HeLa cells interferes with its ability to change alternative splice sites (Rafalska et al., 2004). Indications are that c-Abl could mediate this phosphorylation, at least in HeLa cells, however, more detailed studies are required to elucidate this further. Under high glucose conditions, in human bladder cancer cells, YTHDC1 has been shown to undergo lysine lactylation at K82 and this has been shown to control YTHDC1 protein stability (Dai et al., 2025; Xing et al., 2025). Specifically, lactylation of YTHDC1 is mediated by Alanyl-tRNA synthetase (AARS1) and this directs recruitment of the ubiquitin proteasome system (UPS) degradation pathway through binding of RNF138. Taken together, this links YTHDC1 lactylation to a major metabolic hallmark of cancer, the Warburg effect, which is induced by increased glucose uptake and lactic acid production (Xing et al., 2025).

## 4 YTHDC1 in chromatin

As discussed above, increasing evidence of a role for YTHDC1 in chromatin regulation has been identified. We present an overview of the state of the art below.

### 4.1 Gene silencing and heterochromatin

#### 4.1.1 X chromosome inactivation

**FIGURE 3:**
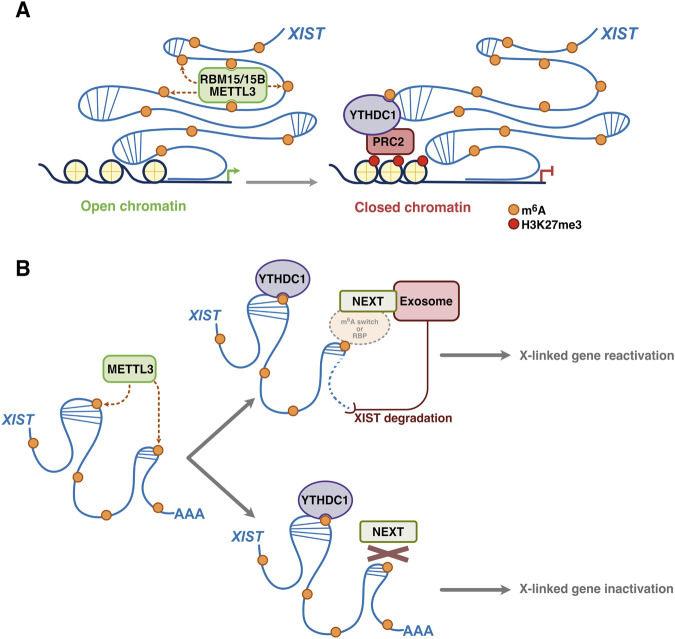
Role of m^6^A/YTHDC1 in X chromosome inactivation. **(A)** m^6^A-modification of *XIST*, mediated by METTL3 following its recruitment by RBM proteins (RBM15/15B), enables the docking of YTHDC1 and the recruitment of silencing complexes such as the Polycomb repressive complex 2 (PRC2), leading to depostition of the repressive mark H3K27me3, chromatin compaction, and transcriptional silencing. **(B)** Under acute depletion conditions (degron system; see text for details), METTL3-dependent m^6^A regulates *XIST* RNA turnover, through the NEXT complex and the nuclear exosome, in XCI. This mechanism occurs independently of YTHDC1 activity, suggesting the involvement of other RNA-binding proteins (RBPs) that may be recruited either directly via m^6^A or through an m^6^A-dependent ‘switch’ that favors local opening of *XIST* RNA secondary structures and facilitates binding of RBPs to adjacent sites. *Adapted from* Patil et al., 2016; Wei et al., 2025 *.*

X chromosome inactivation (XCI) is a mammalian dosage compensation mechanism that ensures balanced expression of X-linked genes between males and females. Intense research over the past decades has clarified XCI mechanisms and dynamics, in particular, the role of the long noncoding RNA, *XIST,* and its protein partners (Alfeghaly and Rougeulle, 2025). Briefly, *XIST* together with specific interacting proteins functions in cis to silence an entire X chromosome. More recently, interest has been sparked as to the role of m^6^A-YTHDC1 in these processes, with contrasting results (Patil et al., 2016; Wei et al., 2025) (Figure 3).

The Jaffrey laboratory identified m^6^A-YTHDC1 as a key component in *XIST* function in XCI (Patil et al., 2016). Indeed, by iCLIP (individual-nucleotide resolution UV crosslinking and immunoprecipitation), *XIST* has been shown to display high levels of m^6^A (around 78 m^6^A sites identified). These sites are positioned by METTL3 targeting to *XIST*, consequent to binding and recruitment to *XIST* by the RBM15 and RBM15B (RNA-binding motif protein 15) proteins. *XIST* RBM15/15B-binding sites were localized adjacent to the *XIST* m^6^A sites (within the DRACH consensus motif) but distant to non-methylated DRACH motifs (Patil et al., 2016). In a screen for *XIST* binding among the YTH domain family members, only YTHDC1 was demonstrated to bind preferentially to m^6^A-modified *XIST* where it promoted *XIST*-mediated transcriptional silencing in an m^6^A-dependent manner. For XCI assays, the authors used male, mouse embryonic stem cells (mESCs) that express *XIST* on the X chromosome in a doxycycline-dependent manner. A female mESC line engineered to express a dox-inducible *XIST* was also used. Silencing was monitored by RNA fluorescence *in situ* hybridization (FISH) against the X chromosome genes *Gpc4* and *Atrx*, at the single nucleus level. Artificial tethering of YTHDC1 to *XIST* rescues *XIST*-mediated silencing upon loss of m^6^A by short interfering RNA (siRNA) against METTL3. Furthermore, YTHDC1 was found to be enriched in the *XIST* nuclear subcompartment, in comparison to autosomal domains, as measured by 3D structured illumination super-resolution microscopy (3D-SIM). These data thus reveal a pathway of m^6^A formation and recognition that is required for *XIST*-mediated transcriptional repression and for assembly of *XIST* silencing compartments in the nucleus (Patil et al., 2016). How YTHDC1 binding to *XIST* leads to gene silencing remains unclear. Hypothesizing that YTHDC1 binding to *XIST* at m^6^A sites might direct the recruitment of *XIST*-dependent silencing complexes at discrete m^6^A-marked XIST regions, the investigators mined published and public proteome interaction data to search for interactions of YTHDC1 with members of *XIST*-dependent silencing complexes (SHARP/SPEN, HDAC3, HNRNPK, HNRNPU, NCOR2 - also known as SMRT-, LBR, PRC1, and PRC2; see a recent excellent review for details: Alfeghaly and Rougeulle, 2025). Interestingly, all were identified as either directly or indirectly interacting with YTHDC1 supporting a model by which YTHDC1 serves as a docking protein for assembly of these proteins on m^6^A-*XIST* thus promoting XCI (Patil et al., 2016). Further mechanistic studies will be required to functionally validate the relevance of these interactions in the setting of the establishment and maintenance of XCI (Figure 3A) (Patil et al., 2016).

In a different approach, utilizing the dTAG-degron system (Nabet et al., 2018) to target METTL3 for acute degradation, the Brockdorff laboratory has shown that *XIST*-mediated silencing is rapidly increased through stabilization of *XIST* (Wei et al., 2025). In this experimental system for XCI, the primary function of m^6^A on *XIST* RNA is thus to promote transcript turnover and removal of METTL3 increases the rate of *XIST*-mediated silencing. The nuclear-exosome-targeting (NEXT) complex was subsequently shown to be essential for *XIST* degradation through a pathway that functions independently of YTHDC1 (Wei et al., 2025). The authors propose two elegant models to explain their findings. In one, it is speculated that an unidentified reader protein interacts with both m^6^A and the NEXT to mediate nuclear RNA degradation. An alternative model proposes that m^6^A in *XIST* acts as a ‘switch’ to indirectly promote the recruitment of *XIST* RBPs that consequently promote NEXT exosome complex access and activity on *XIST*. Candidate RBPs in the latter setting could be hnRNPC and hnRNPA2B1 proteins that have been previously reported to function indirectly through an ‘m^6^A switch’ mechanism (Figure 3B) (Liu et al., 2015; Porman et al., 2022).

Despite some conflicting results, the above studies clearly implicate m^6^A-YTHDC1 in XCI. The work also underscores the importance of experimental design (chronic versus acute depletion of m^6^A levels, for example) when studying the role of m^6^A in RNA-dependent transcriptional silencing or activation processes. It is possible that chronic versus acute perturbation of m^6^A-YTHDC1 mediates distinct functional outputs in physiological or disease settings. The latter merits careful exploration and findings will be of keen interest in the field of XCI and beyond, particularly in cancer research where perturbation of nuclear non-coding RNA expression and function - including for *XIST* (increased blood cancer risk, at least in mice) (Yildirim et al., 2013) - is known to drive disease processes (Li and Fu, 2019; Xie and Dean, 2025).

#### 4.1.2 Activity of endogenous retroviral elements

**FIGURE 4:**
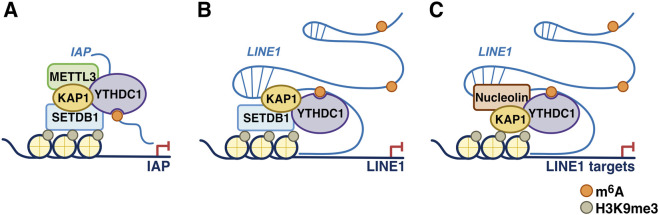
m^6^A-YTHDC1 in transposable elements silencing. **(A)** m^6^A deposition by METTL3 on intracisternal A particle (*IAP*) RNAs promotes the recruitment of YTHDC1 and chromatin silencing factors such as KAP1/TRIM28 and SETDB1, resulting in H3K9me3 deposition and transcriptional silencing of *IAP* loci in mESCs. **(B)** m^6^A modification of *LINE1* RNA leads to the recruitment of YTHDC1-KAP1/TRIM28-SETDB1 complexes, promoting heterochromatin formation and transcriptional repression of *LINE1* elements. **(C)** m^6^A deposition on a subset of *LINE1* RNAs contributes to their scaffold function, notably through the recruitment of YTHDC1 and Nucleolin/KAP1/TRIM28, thereby modulating the expression of *LINE1* and *LINE1*-rich genes. Adapted from He and Lan, 2021.

As discussed above, METTL3 mediates the N6-methyladenosine (m^6^A) methylation of mRNA, which affects the stability of mRNA and its translation into protein. Similar to METTL3 and m^6^A-YTHDC1 activity in the control of enhancer activity and transcription, their role in control of heterochromatin homeostasis, is also increasingly described. Mechanisms appear diverse and may depend on cell context and RNA secondary structure (Chelmicki et al., 2021; Chen et al., 2021; Liu et al., 2021; Xu et al., 2021) (Figure 4).

In one study, METTL3 has been shown to regulate heterochromatin in mESCs (Xu et al., 2021), the integrity of which is critical for silencing retroviral elements and for mammalian development. Specifically, METTL3 was found to predominantly localize to the intracisternal A particle (IAP)-type family of endogenous retroviruses (a murine-specific ERV family), especially the IAPEz subtype. In *Mettl3* KO mESCs, the deposition of heterochromatin marks onto METTL3-targeted IAPs was found to be impaired, and this was associated with upregulation of *IAP* transcription (rather than increased *IAP* RNA stability), suggesting that METTL3 is important for the integrity of IAP heterochromatin. It is to be noted that proliferation or other defects were not reported for the *Mettl3* KO mESCs, unlike what is seen in *Ythdc1* KO (see below) (Liu et al., 2021). Interestingly, RNA transcripts derived from METTL3-bound IAPs were found to be associated with chromatin and m^6^A-methylated, as measured by chromatin isolation by RNA purification with sequencing (ChIRP–seq) and methylated RNA immunoprecipitation (MeRIP), respectively. These m^6^A-marked transcripts are bound by YTHDC1, which through interaction with METTL3, promotes further association of METTL3 with chromatin, thus creating a positive feedback loop whereby each protein reinforces each other’s recruitment in chromatin. Furthermore, in this study system, METTL3 was also found to interact physically with the histone 3 lysine 9 (H3K9) tri-methyltransferase SETDB1 and its cofactor KAP1/TRIM28, and to be required for their localization to IAPs, in a mechanism that appears not to require METTL3 catalytic activity. Taken together, these findings reveal that METTL3-catalysed m^6^A modification of RNA is important for the integrity of IAP heterochromatin via m^6^A-YTHDC1 and recruitment of SETDB1-KAP1/TRIM28, thus revealing a novel mechanism of heterochromatin regulation in mammals (Xu et al., 2021) (Figure 4A).

A second study has directly investigated the function of YTHDC1 in transposable elements (TE) silencing by using a conditional knockout (cKO) strategy, targeting the YTH domain, in mESCs (Liu et al., 2021). Consistent with the early embryonic lethal phenotype of *Ythdc1* KO in mice, *Ythdc1* cKO mESCs showed impaired proliferation and apoptosis after only a limited number of passages (unlike *Mettl3* KO mESCs, discussed above). Also, *Ythdc1* cKO mESCs were enriched for expression of genes seen in the embryonic 2-cell state (2C), including those of the *Zscan4* family, *Dux* and the transposable element *MERVL*, indicating induction of the embryonic 2C-like state transition, and thus a role for YTHDC1 in ES cell identity. Wildtype YTHDC1 but not YTH m^6^A-binding-site mutants of YTHDC1 (K362A/S363A/N364A, W378A and W429A) could rescue the activation of the 2C-like program, indicating that the 2C-like transition is initiated in an m^6^A-YTHDC1-dependent manner. Furthermore, by epigenomic assays, it could be shown that YTHDC1 binds m^6^A-modified TEs, including LINE1 and ERVs (such as IAPs) in chromatin (Liu et al., 2021). Biochemical analyses revealed a physical interaction between YTHDC1 and the histone H3 lysine 9 trimethylase, SETDB1, suggesting that YTHDC1 could recruit SETDB1, upon binding to target TEs. In keeping with this, YTHDC1 and SETDB1/H3K9me3-marked TEs were found to significantly overlap, and depletion of YTHDC1 and SETDB1 leads to a reduction in H3K9me3 methylation at the target TEs. The mechanisms described in the above *Ythdc1* cKO model appear thus to differ from those seen in *Mettl3* KO mESCs, suggesting that m^6^A-YTHDC1 signaling mechanisms in heterochromatin may vary according to mESC cell state, across TE classes, and possibly according to baseline m^6^A methylase activity or m^6^A landscapes. These observations collectively indicate a co-regulation of TEs by m^6^A-YTHDC1 and SETDB1-mediated H3K9me3 in early development (Liu et al., 2021) (Figure 4B).

As discussed above, m^6^A-YTHDC1 has been found to play indispensable roles in self-renewal and differentiation potency in early development and this depends on its ability to silence transcription of TEs, such as LINE1 and ERVs, in part, by recruitment of histone methyltransferase activity to chromatin. A third way in which m^6^A-YTHDC1 can regulate heterochromatin is through modulation of the scaffold function of *LINE1* which plays essential roles in mESCs and pre-implantation embryos (Percharde et al., 2018; Chen et al., 2021). Indeed, in mESCs, *LINE1* RNA has been found to act as a nuclear RNA scaffold that recruits Nucleolin and KAP1/TRIM28 to repress *Dux*, the master activator of a transcriptional program specific to the 2-cell embryo (Percharde et al., 2018). In parallel, *LINE1* RNA mediates binding of Nucleolin and KAP1/TRIM28 to rDNA, promoting rRNA synthesis and ES cell self-renewal. Thus, in embryos, *LINE1* RNA is required for *Dux* silencing, synthesis of rRNA and exit from the 2-cell stage, thereby establishing an essential partnership between *LINE1* RNA, Nucleolin, KAP1/TRIM28 and peri-nucleolar chromatin in the regulation of transcription, developmental potency and ES cell self-renewal (Percharde et al., 2018). It has since been uncovered that the *LINE1* scaffold function depends on m^6^A-YTHDC1 (Chen et al., 2021). Specifically, YTHDC1 has been found to recognize m^6^A sites in a subset of m^6^A-marked *LINE1* RNAs in the nucleus, and this function allows formation of the *LINE1*-Nucleolin partnership and the chromatin recruitment of KAP1/TRIM28. The *LINE1* RNA species involved in this scaffold complex were identified as a subpopulation of *LINE1* RNAs on chromatin that contain METTL3-insensitive m^6^A sites and that are targeted by YTHDC1, resulting in their stabilization (rather than destabilization, as described above). Intriguingly, in this latter subset of *LINE1* RNA, m^6^A appeared to be centered on a sequence pattern highly resembling the ACAGAGA motif that is recognized by METTL16 in U6 snRNA. Interestingly, the establishment of H3K9me3 on 2C-related retrotransposons was found to be interrupted in YTHDC1-depleted mESCs and inner cell mass cells, coincident with increased transcriptional activity (Chen et al., 2021). Interestingly, epigenomic explorations revealed that silencing of LINE1-enriched genes was also disturbed in the latter conditions and this was correlated to reduced KAP1/TRIM28 recruitment, although further mechanistic investigations were not done on the latter targets. Taken together, this work establishes a novel role for m^6^A-YTHDC1 in regulation of the *LINE1*-Nucleolin-KAP1/TRIM28 scaffold complex and its function in heterochromatin assembly at TE elements, thus providing a new model for the RNA-chromatin cross-talk (Chen et al., 2021) (Figure 4C).

Homeostatic regulation of ERVs is achieved by surveillance at different steps of the ERV life cycle. Notably, chromatin-based silencing by DNA methylation and histone modifications and post-transcriptional control through RNA editing and RNA interference have been extensively characterized (Zamudio and Bourc’his, 2010). In this respect, and although outside of the direct scope of the present review, it should be noted that an additional mechanism for post-transcriptional clearance of TE transcripts have been described in mESCs and this depends on m^6^A deposition by METTL3 and binding by YTHDF2 which leads to their targeting for degradation, most probably in the cytoplasm, similarly to mRNAs (Chelmicki et al., 2021). Of note, in the latter study, this post-transcriptional control mechanism was uncovered by using a degron strategy to achieve rapid depletion of both METTL3 and METTL14 in mESCs. Post-transcriptional, m^6^A-dependent mechanisms for ERV suppression may operate alongside m^6^A-YTHDC1 chromatin-dependent silencing mechanisms or may prove crucial when heterochromatin silencing is less stringent (Chelmicki et al., 2021).

### 4.2 Enhancer and transcriptional regulation

Pervasive transcription of multiple non-coding RNAs is a characteristic of mammalian genomes. Of the non-coding transcripts that are produced, chromatin-associated regulatory RNAs (also referred to as chromatin-associated non-coding RNAs) (carRNAs) are attracting considerable interest for their role in shaping chromatin architecture and transcriptional control *in cis* and *in trans* (Li and Fu, 2019; Xie and Dean, 2025). carRNAs can be divided into 3 functional groups: promoter associated RNAs (paRNAs), enhancer RNAs (eRNAs) and RNA transcribed from transposable elements (TE) (repeat RNAs) (Liu et al., 2020). Two recent studies have provided evidence of a regulatory role for m^6^A-YTHDC1 in carRNA stability and function in chromatin, with some contrasting results (Liu et al., 2020; Lee et al., 2021).

**FIGURE 5:**
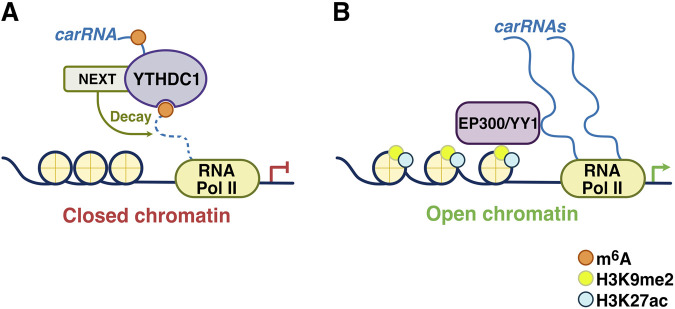
Regulation of local chromatin states by carRNAs and m^6^A-YTHDC1. **(A)** m^6^A deposition on chromatin-associated RNAs (carRNAs) promotes their decay through m^6^A-YTHDC1-mediated recruitment of the NEXT complex and a closed chromatin state at these genomic loci. **(B)** In the absence of m^6^A modification, carRNAs are stabilized and can promote the local deposition of activating histone marks (H3K9me2 and H3K27ac), resulting in an open chromatin state through the recruitment of factors such as the histone acetyltransferase EP300 and the transcription factor YY1. Adapted from Liu et al., 2020.

In the study by Liu et al., knockout of *Mettl3* or *Ythdc1* in mESCs increased chromatin accessibility and downstream transcription, in an m^6^A-dependent manner (Figure 5). Consistent with this, both H3K4me3 and H3K27ac, two histone marks associated with active transcription, were increased upon METTL3 and YTHDC1 depletion, in keeping with a nuclear regulatory role for RNA m^6^A. In this system, METTL3 deposits m^6^A modifications on carRNAs, including paRNAs, eRNAs, and repeat RNAs. Quite strikingly, 80% of eRNAs associated with super-enhancers are m^6^A-modified (Liu et al., 2020). YTHDC1 facilitated the decay of a subset of carRNAs, mostly long interspersed element-1 family elements (LINE1), through NEXT-mediated nuclear degradation, components of which (RBM7, ZCCHC8) were shown to physically interact with YTHDC1 (Figure 5A). Importantly, in *Ythdc1* cKO mESCs, m^6^A abundance on mRNA was not notably altered, suggesting unique regulatory functions for m^6^A-YTHDC1 in carRNAs. Consistently, reducing m^6^A methylation of selected carRNAs, by METTL3 depletion or site-specific m^6^A demethylation, results in increased carRNA levels, open chromatin state and downstream transcription (Figure 5B). Functionally, the latter was attributed to increased binding of the histone acetyltransferase EP300, and YY1 at regions that lose m^6^A-marked carRNAs (Liu et al., 2020). The genomic regions with both m^6^A-marked carRNAs and binding of both EP300 and YY1 showed the greatest increase in acetylated H3K27, compared with regions with just m^6^A or just EP300/YY1 binding, upon *Mettl3* KO. Interestingly, chromatin immunoprecipitation and sequencing (ChIP-seq) of JARID2, a component of the Polycomb repressive complex 2 (PRC2), revealed increased JARID2 binding at chromatin regions associated with m^6^A-marked carRNAs, in *Mettl3* KO compared to control conditions. Taken together, in mESCs, m^6^A-regulated carRNAs may stabilize open chromatin not only by facilitating recruitment of factors such as EP300/YY1 but by repelling PRC2 binding (Liu et al., 2020). The latter hypothesis is in keeping with mechanistic studies that have revealed RNA-dependent inhibition of PRC2 recruitment to chromatin (Wang et al., 2017). Taken together, this study reveals a functional crosstalk between carRNA m6A methylation and chromatin state in mESCs.

By using a highly sensitive method called methylation-inscribed nascent transcripts sequencing (MINT-seq), Lee *et al* characterized the nascent m^6^A RNA landscape in three widely used human cell line models (MCF7, K562 and HeLa, respectively), and revealed a function for m^6^A-YTHDC1 in eRNA function (Lee et al., 2021) albeit with some contrasting results compared to those seen in *Ythdc1* cKO mESCs described above (Liu et al., 2020). Briefly, MINT-seq revealed selective m^6^A deposition on nascent RNAs produced by transcription regulatory elements, including promoter upstream antisense RNAs (uaRNAs) and eRNAs. The m^6^A marks positively correlated with transcript length, presence of consensus m^6^A motifs, and RNA abundances. As expected for eRNAs, strong cell-type specificity for m^6^A modification patterns was seen. In MCF7 cells (breast cancer cell line), without and with estrogen stimulation, for rapid enhancer activation, m^6^A-eRNAs were found to mark highly active enhancers. YTHDC1 was found to transcriptionally regulate and bind to m^6^A-eRNAs, at m^6^A-marked sites in chromatin and to facilitate their phase separation into liquid-like condensates, with BRD4, in a manner dependent on the YTHDC1 C-terminal IDR, thus linking m^6^A-eRNA-YTHDC1 to BRD4-dependent super-enhancer activity and transcription. Consistent with this, by ChIP-seq, YTHDC1 was found to bind 1760 sites in the genome of which 61.1% corresponded to putative enhancers, as defined by p300 binding sites (intergenic and intronic) and a strong correlation was seen between YTHDC1 and BRD4 binding at m^6^A-eRNA-marked enhancers. Furthermore, YTHDC1 depletion, by siRNA, diminished BRD4 condensates (by microscopy) and BRD4 recruitment to enhancers (ChIP-seq), resulting in reduced enhancer activity and gene activation. Taken together this study further implicates YTHDC1-m^6^A-eRNA and phase separation capacities in enhancer activation and transcription.

The reasons for the differences in results seen in the above studies are not yet clear. However, as for XCI, it is likely that m^6^A-YTHDC1 signaling dynamics vary under different experimental conditions (constitutive or conditional genetic loss compared to transient, acute knockdown, for example) and across different targets, in species-specific and cell context-dependent manner. This could be an important focus for future work regarding m^6^A-YTHDC1 in chromatin state regulation in normal and disease processes.

An alternate mechanism by which m^6^A-YTHDC1 can promote transcriptional activation has been shown to involve interaction of YTHDC1 with chromatin modifiers that can destabilize heterochromatin directly at gene promoters. In a study by Li *et al*, YTHDC1 has been shown to bind to m^6^A-marked nascent RNA at chromatin and to favor removal of the repressive H3K9me2 mark by physically interacting with and recruiting the histone H3 lysine 9 demethylase KDM3B, to these m6A-associated chromatin regions, thus promoting gene expression (Li et al., 2020). This mechanism was uncovered by using a screening system comprising a tetracycline-inducible reporter in human Flp-In HEK293 cells to directly investigate the effects of m^6^A on histone modifications. Briefly, this system comprised two plasmids containing tetracycline-inducible reporter transcripts, one with the m^6^A modification motif, GGAC, and the second without (GGTC), as a control. Using this reporter strategy, the authors found a marked decrease in H3K9me2 (about 4-fold) in the reporter cells compared to controls. Slight increases in H3K27ac and H3K27me3 were also seen which would contrast with results seen in neural stem cells upon cKO of *Mettl14* (reduced m^6^A, associated with increased H3K27ac and H3K4me3 as well as decreased H3K27me3) (Wang et al., 2018). However, the two experimental systems and observed cellular phenotypes are markedly different and are thus difficult to compare. The changes seen in H3K9me2, in HEK293 cells, were not associated to changes in expression of enzymes responsible for regulation of this histone modification and could be reversed by METTL3 depletion by siRNA, thus pointing to a connection between histone marks and m^6^A. Consistently, H3K9me2 decreases could be rescued by wildtype METTL3 but not by the catalytically inactive METTL3 D395A mutant. Similarly, knockdown of the m^6^A demethylases, FTO and ALKBH5, resulted in increased m^6^A and decreased H3K9me2, mostly in gene bodies near to or overlapping m^6^A sites compared to non-m^6^A sites.

Raising the hypothesis that this might be explained by differential recruitment of a histone lysine demethylase (KDM) activity to the affected loci, more detailed epigenomic and mechanistic analyses were done in 2 cell models (HEK293 and mESCs), focusing on KDM3B for which protein expression was seen to be highly correlated to METTL3 (at least in mESCs). ChIP-seq revealed co-occupancy of chromatin by METTL3 and KDM3B in up to 70% of sites. KDM3B peaks overlapped m^6^A sites (RIP-seq) and this was strongly correlated to METTL3 peaks and to RNA polymerase II peak summits (ChIP-seq), thus supporting co-transcriptional recruitment of KDM3B to target loci. In keeping with this, treatment with the RNA polymerase II inhibitor, 5,6-dichlorobenzimidazole-1- β-D-ribofuranoside (DRB), significantly decreased the abundance of chromatin-bound KDM3B. Subsequently, testing whether m^6^A reader proteins might recruit KDM3B to chromatin, YTHDC1 (but not HNRNPA2B1 or YTHDF2) was found to interact with KDM3B and to co-localize with KDM3B in the nucleus (in HEK293 and mESCs). YTHDC1 knockdown reduced KDM3B recruitment to m^6^A-marked chromatin, as assessed by ChIP-seq, thus mimicking what is seen in METTL3 activity-deficient conditions (METTL3 D395A). Targeted recruitment of YTHDC1 to selected loci by using dPspCas13b-YTHDC1 and gRNA was sufficient to recruit KDM3B and to decrease H3K9me2 at the target loci. Taken together, these results establish a model in which modification of nascent RNA by the m^6^A methylation complex recruits KDM3B through the reader protein YTHDC1, thus locally decreasing levels of H3K9me2.

Finally, although a specific role for YTHDC1-m^6^A was not investigated, the study by Wang *et al* revealed that m^6^A contributes to the destabilization of transcripts encoding histone-modifying enzymes and complexes, including KDM6B, CBP, and P300, thereby influencing activating chromatin marks such as H3K27ac (Wang et al., 2018). At least in leukemia, this mechanism may operate to increase expression of chromatin modifiers such as KMT2C (Li et al., 2025).

### 4.3 Chromatin state in development and the cellular response to stress

#### 4.3.1 Development and bivalent chromatin

Bivalent chromatin defines a paradoxical chromatin state, characterized by the co-occurrence of activating and repressing chromatin marks (such as H3K4me3 and H3K27me3, respectively), at the promoters of developmental genes (Macrae et al., 2023). This bivalent chromatin state is thought to poise important regulatory genes for expression or repression during cell-lineage specification (Macrae et al., 2023). Until recently (Dong et al., 2025), the potential role of epitranscriptome-mediated-regulation of bivalent chromatin had remained uninvestigated.

Dong *et al* used an elegant human iPSC-to-lung progenitor (LP) model to investigate the role of m^6^A-YTHDC1 in early lung development (Dong et al., 2025). Specifically, the dynamics and stage-specific m^6^A landscape in human iPSCs, definitive endoderm (DE), anterior foregut endoderm (AFE), and LPs were characterized throughout the differentiation process. Briefly, by dot blot analysis in total RNA, m^6^A levels were found to globally increase over the differentiation stages. This was further confirmed by picogram-scale m^6^A RNA immunoprecipitation and sequencing (picoMeRIP-seq) which revealed 13,906 m^6^A peaks in iPSCs, 17,200 in DEs, 18,017 in AFEs, and 20,531 in LPs. Analysis of the RNA species revealed that the majority of m^6^A peaks were detected in protein-coding transcripts, followed by long non-coding RNAs (lncRNAs), pseudogenes, and small RNAs. Across all four developmental stages, the majority of m^6^A peaks in mRNAs were found within the coding sequence (CDS) and 3′UTR, particularly near stop codons, with the most predominant changes occurring in the iPSCs to DE transition (see above) (Dong et al., 2025). Further analysis revealed specific upregulation of the m^6^A writer RBM15B, in the pluripotency (iPSCs) to DE transition. By knockdown studies, RBM15B was found to drive elevated m^6^A levels in differentiated cells, in particular at key bivalent genes of which *GATA4*, *GATA6*, and *EOMES*. Concurrently, loss of YTHDC1 was found to alleviate PRC2-mediated transcriptional silencing of these targets. In keeping with this, a physical interaction between YTHDC1 and SUZ12/EZH2 was found in iPSCs and this remained largely unchanged in DE cells. Of note, a reduction of YTHDC1 protein levels was observed in DE cells compared to iPSCs. Furthermore, YTHDC1 was found to bind mRNAs and promoters of PRC2 target genes, such as *GATA4*, *GATA6*, and *EOMES*, in iPSCs. YTHDC1 thus plays dual functions as an m^6^A reader and a scaffold protein to recruit PRC2 to the promoters of bivalent genes. Furthermore, a switch between m^6^A readers YTHDC1 and IGF2BP2 was found to orchestrate transcription and RNA stability regulation at these bivalent genes, thereby directing endoderm differentiation. Overall, this study highlights the role of m^6^A-centered regulatory mechanisms in the early stages of embryonic lung development, in particular through establishment and resolution of the bivalent chromatin state at key developmental genes (Dong et al., 2025).

#### 4.3.2 Chromatin and transcriptional response to stress

The highly conserved heat shock (HS) response can be triggered by multiple external stresses that include heat, hypoxia, protein aggregation, and heavy metals, as well as by certain physiopathological states, and it directs the rapid and global reprogramming of transcription at genes and enhancers (Gomez-Pastor et al., 2018; Vihervaara et al., 2018).

The Verdel laboratory has shown that human YTHDC1 mostly associates with the chromatin fraction and that HS induces a redistribution of YTHDC1 across the genome, including to heat-induced heat shock protein (HSP) genes (Timcheva et al., 2022). YTHDC1 binding to m^6^A-modified *HSP* transcripts was found to occur co-transcriptionally and to promote expression of HSPs. In parallel, hundreds of genes showing increased YTHDC1 occupancy during HS display YTHDC1- and m^6^A-dependent intron retention in the corresponding transcripts. Among the latter, were *TAF1D* (TATA box binding protein associated factor, RNA polymerase I subunit D), *DNAJB9* (DNAJ heat shock protein member) and *CLK1* (CDC-like kinase 1). Interestingly, CLK1 has been implicated in splicing control by intron retention. In later stages of the HS response, YTHDC1 was found to relocate and concentrate within nuclear stress bodies (nSBs) where it bound to m^6^A-modified *SATIII* non-coding RNAs, a class of repeat-derived RNAs which is induced upon activation of the master HS response transcription factor, HSF1. These findings reveal that YTHDC1 plays a central role in a chromatin-associated m^6^A-based reprogramming of gene expression during HS (Timcheva et al., 2022). Furthermore, the data support a model whereby the subsequent and temporary sequestration of YTHDC1 within nSBs calibrates the timing of this stress-induced YTHDC1-dependent gene expression reprogramming.

### 4.4 Genome stability

A number of studies now implicate m^6^A-YTHDC1/YTHDC1 in genome stability through involvement in repair of both physiological (class switch recombination in B cells, for example) (Nair et al., 2021) and genotoxic stress-induced DNA damage (Zhang et al., 2020; Tsao et al., 2025) and in the control of p53 response pathways (Elvira-Blazquez et al., 2024).

#### 4.4.1 Immunoglobulin gene class switch recombination and B cell development

The production of mature B cells requires programmed, somatic recombination events at immunoglobulin gene loci that generate functional and diverse antibody isotypes. Two key processes can be distinguished: V(D)J recombination and class switch recombination (CSR) (McConnell and Casadevall, 2025). The latter determines the immunoglobulin heavy chain constant region isotypes that will be expressed thus allowing the production of IgM, IgD, IgG, IgE or IgA through a recombination process that involves AID (Activation-induced Cytidine Deaminase), non-coding RNAs produced at switch regions and the RNA exosome complex (McConnell and Casadevall, 2025).

In a recent exploration of this process regarding the role for m^6^A and m^6^A readers, METTL3-catalyzed m^6^A modification was found to drive recognition and 3′ end processing of *SμGLT* by the RNA exosome, thereby promoting CSR and suppressing chromosomal translocations (Nair et al., 2021). This recognition is driven by interaction of the MPP6 adaptor protein with YTHDC1. MPP6 and YTHDC1 promote CSR by recruiting AID and the RNA exosome to actively transcribed *SμGLT*. Direct suppression of m^6^A modification of *SμGLT*, or depletion of YTHDC1, reduces CSR. To address more specifically the role of m^6^A in B cell development, the authors constructed a *Mettl3* ^*f/f*^ *Mb1* ^*cre/+*^ mouse model in which the *Mettl3* gene is deleted in the bone marrow, early in B cell development. METTL3 depletion in bone marrow-developing B cells was found to lead to a clear block at the pro-B cell to pre-B cell stage with a marked decrease in pre-B cells. B cell populations in the periphery, such as follicular and marginal zone B cells in the spleen, and B cells in germinal centers (GCs) present in Peyer’s patches, were also significantly decreased (Nair et al., 2021). Taken together, this points to a role for METTL3 functions in B cell development and survival in both the bone marrow and the GC reaction. In this model, a role for METTL3 was found in suppression of off-target CSR/IgH-associated aberrant DNA breaks and thus genomic instability. Taken together, these results suggest coordinated and central roles for MPP6, m^6^A modification, and m^6^A reader proteins in controlling long noncoding RNA processing, DNA recombination and development in B cells (Nair et al., 2021).

#### 4.4.2 p53 signaling

Loss of p53 tumor suppressor functions, mostly by genetic mechanisms, is described in the vast majority of cancers, thus identifying improved therapy for p53 defective cancers as a major therapeutic goal (Peuget et al., 2024). A recent study has identified a major role for YTHDC1 in control of p53 transcription and in splicing of p53-response gene transcripts (Elvira-Blazquez et al., 2024).

Briefly, the Huarte laboratory utilized an RBP/RNA-modifier-focused CRISPR-Cas9 knockout screen to identify factors controlling the activity of a p53-dependent transcriptional response. Specifically, the authors devised a p53 response reporter system comprising the p21 promoter coupled to mVenus in A549 lung cancer cells that had been engineered to express SpCas9 endonuclease. These cells were transduced by a custom lentiviral-packaged CRISPR library designed to target 407 genes that encode proteins with known RNA binding and/or modifier activity. Following Nutlin-3a treatment, a potent inhibitor of Mdm2-mediated p53 degradation, to stabilize p53 and thereby induce p53 response at the p21 reporter and globally in the cells, cells were sorted by reporter activity (attenuated or increased mVenus, respectively). RNAseq analysis was used to identify known and novel targets that either increased or attenuated p53 response. Among the top hits that attenuated p53 response was *YTHDC1* which was found to reduce reporter activity coincident with significant decreases in p53 protein levels. Mechanistically, these effects could be attributed to m^6^A-independent functions of YTHDC1 in transcriptional control of *TP53*. Indeed, knockdown or CRISPR-Cas9 KO of *YTHDC1* decreased *TP53* mRNA and pre-mRNA, as assessed by qRT-PCR, in various cell lines corresponding to different cancerous tissue types (A549 (lung); MCF7 (breast), HeLa (uterus/cervix) and HCT116 (colon)). METTL3 depletion or inhibition did not cause a decrease of either mature or pre-mRNA *TP53* RNA levels, suggestive of an m^6^A-independent mechanism. Considering that transcription is a complex stepwise process comprising multiple steps, including the recruitment and assembly of the machinery responsible for initiation, pause-release, elongation, and termination, the authors investigated pause-release mechanisms as a possible explanation. Indeed, polymerase pausing represents a critical, tightly regulated checkpoint of the transcription process that upon disruption can lead to cellular dysfunction, cancer or aging (Compe and Egly, 2021; Gyenis et al., 2023). Hypothesizing that YTHDC1 depletion may specifically alter transcription dynamics, the authors performed RNA Pol II ChIP-seq in both *YTHDC1*-knockdown and siRNA control A549 cells. Additionally, YTHDC1 ChIP-seq and chromatin-associated RNA crosslinked immunoprecipitation (CLIP-seq), to further interrogate the relationship between transcription and YTHDC1 DNA and RNA binding genome-wide, respectively. Briefly, these assays revealed a crucial role for YTHDC1 in transcription elongation at the *TP53* promoter and genome-wide at sites associated with m^6^A or non-m^6^A transcripts. Indeed, dramatic increases in paused RNA Pol II, calculated as an RNAPII pausing index value (log_2_ RNAPII promoter density/RNAPII gene body density) were seen in YTHDC1-depleted cells versus controls, comparable to what is seen when comparing wildtype and knockout cells of the elongation factor NELF (Wu et al., 2022). Consistently, this increased RNA Pol II pausing was correlated to reduced YTHDC1 at the affected transcription start sites (TSS) by ChIP-seq but not by CLIP-seq, thus suggesting a role for YTHDC1 in transcriptional control at the DNA/chromatin level. A caveat, however, is that expression of either wildtype or YTH-inactive YTHDC1 did not rescue RNA Pol II pausing at *TP53* in YTHDC1-deficient A549 cells. Other rescue methods, such as direct tethering strategies, were not attempted. There is thus a need for more mechanistic work to fully unravel the role of YTHDC1 in transcription pause release, specifically at key targets such as *TP53*.

In addition to the effects of YTHDC1 on *TP53* transcription, and in keeping with its known functions in splicing, YTHDC1 was also found to directly regulate splicing and protein expression of several p53-regulated DNA damage response (DDR)-related pathway members, such as ATR, BIRC6 and SETX. Specifically, depletion of YTHDC1 was associated with aberrant intron retention and reduced protein expression of these targets. These splicing defects could be rescued by wildtype YTHDC1, but not m^6^A-binding defective YTHDC1 mutants (W377A/W428A), consistent with m^6^A-dependent mechanisms. Impaired DDR was a predicted consequence of mis-splicing and reduced expression of ATR, BIRC6 and SETX. In keeping with this, cisplatin treatment of YTHDC1-depleted A549 cells resulted in increased DNA damage, reduced proliferation and tumorigenicity.

Taken together, this work establishes a role for YTHDC1 in regulation of p53 DDR by both m^6^A-dependent and -independent mechanisms, the latter operating in chromatin and directly affecting *TP53* transcription pause-release (Elvira-Blazquez et al., 2024).

#### 4.4.3 DNA double strand break repair

Beyond a role in the induction of the p53 response pathway through transcriptional control of p53 and regulation of DDR factor splicing and expression, m^6^A-YTHDC1 has been implicated in the unscheduled DNA double strand break (DSB) repair pathway itself (Zhang et al., 2020). Briefly, it has been shown that METTL3 is activated by ATM-mediated phosphorylation at S43 (Zhang et al., 2020). Phosphorylated METTL3 is localized to DNA damage sites, where it deposits m^6^A on DNA damage-associated RNAs. Interestingly, these RNAs recruit YTHDC1 creating a METTL3-m^6^A-YTHDC1 signaling axis that modulates accumulation of DNA-RNA hybrids at DSB sites, leading to recruitment of RAD51 and BRCA1 for homologous recombination (HR)-mediated repair. Consistent with this, METTL3-deficient cells show defective HR, accumulation of unrepaired DSBs, and genome instability. Accordingly, depletion of METTL3 was found to enhance the sensitivity of cancer cells to DNA damage-based therapy. These findings uncover the function of METTL3 and m^6^A-YTHDC1 in HR-mediated DSB repair, with potential implications for cancer therapy (Zhang et al., 2020).

#### 4.4.4 RNA-induced DNA damage

It is known that certain environmental toxins and chemotherapeutics are nucleic acid-damaging agents, inducing adducts in DNA and RNA. While most of these adducts occur in RNA, the consequences of RNA damage are largely unexplored. A recent report has revealed that nuclear RNA damage can result in loss of genome integrity in human cells (Tsao et al., 2025). Specifically, YTHDC1 has been found to regulate alkylation damage responses with the THO complex (THOC). In addition to its established binding to m^6^A, YTHDC1 binds to chemically induced N1-methyladenosine (m^1^A). Without YTHDC1, cells have greater alkylation damage sensitivity and increased DNA breaks, which are rescued by an RNA-specific dealkylase. These RNA-damage-induced DNA breaks (RDIBs) depend on R-loop formation, and their conversion to DNA breaks by the nuclease, XPG. Strikingly, in the absence of YTHDC1 or THOC, a nuclear RNA m^1^A methyltransferase is sufficient to induce DNA breaks. These results provide mechanistic insight into how damaged RNAs can impact genomic integrity and place YTHDC1 as a sensor of this damage via alternate methyladenosine mark binding (Tsao et al., 2025).

## 5 YTHDC1 and blood cancers

Altered YTHDC1 expression, at the RNA and/or protein levels, is seen in numerous solid cancers and in leukemia where it is implicated in oncogenic or tumor suppressor functions and has shown potential as a prognostic and theranostic biomarker (Barbieri and Kouzarides, 2020). Although functional studies investigating the mechanisms that drive aberrant YTHDC1 expression and exploring downstream consequences remain sparse, insights are arising. In the following sections we summarize key mechanistic findings from selected studies in blood cancers (Table 1).

**TABLE 1:** Summary of studies regarding m^6^A-YTHDC1 in human blood cancer**s**.

Blood cancer type	Strategy leading to identification of YTHDC1 as a target of interest	Leukemia cell phenotypes and molecular mechanisms	Oncogene/TSG*	Clinical	Reference
AML	CRISPR-Cas9 screen	Reduced AML cell survival and proliferation in *YTHDC1* CRISPR KO cells Increased *YTHDC1* expression in AML samples Increased abundance of nuclear YTHDC1-condensates m^6^A-YTHDC1-dependent stabilization of oncogene transcripts (*MYC*) within YTHDC1 condensates through protection against degradation by the NEXT or PAXT complexes	Oncogenic	Poor prognosis; YTHDC1-DEG gene signature (containing up or downregulated genes) (TCGA - AML)	Cheng et al. (2021)
AML	Expression analysis in AML patient data (TCGA and primary patient samples compared to normal controls)	Increased expression of *YTHDC1* in AML (TCGA and primary patient samples) compared to normal controls Heterozygous KO or KD *YTHDC1*; reduced leukemia burden and leukemia stem cell functions *in vitro* and *in vivo* KD of *YTHDC1* in primary AML cells; increased apoptosis, differentiation m^6^A-YTHDC1 regulates MCM4 expression and facilitates MCM complex–mediated DNA replication	Oncogenic	Poor survival (TCGA - AML)	Sheng et al. (2021)
AML	Screen for candidate m6A methylase chromatin recruitment factors in epigenomics ENCODE data from K562 (CML) compared to HepG2 cell lines	RBFOX2, recruits RBM15, a m^6^A methylase complex member, to facilitate methylation of promoter-associated RNAs RBM15 physically interacts with YTHDC1 and recruits PRC2 to the RBFOX2-bound loci for chromatin silencing and transcription suppression Downregulation of *RBFOX2* inhibits survival/proliferation of acute myeloid leukemia cells and promotes their myeloid differentiation RBFOX2 is required for self-renewal of leukemia stem/initiation cells and AML maintenance RBFOX2/m^6^A/RBM15/YTHDC1/PRC2 axis plays a critical role in AML	Oncogenic signaling	Poor survival (RBFOX2) (TCGA - AML)	Dou et al. (2023)
B-ALL	Expression analysis	Increased *YTHDC1* expression in treatment refractory B-ALL KD of *YTHDC1* - Attenuated B-ALL cell proliferation and cell cycle progression *in vitro*, and prolonged survival of mice in the human B-ALL xenograft model - Increased accumulation of endogenous and chemotherapeutic agent-induced DNA damage YTHDC1 binds to and stabilizes m^6^A-modified *KMT2C* mRNA and promotes expression of DDR genes by deposition of ‘active’ histone mark (H3K4me3)	Oncogenic	NA	Li et al. (2025)

Abbreviations: AML (acute myeloid leukemia), B-ALL (B-cell acute lymphoblastic leukemia), CML (chronic myeloid leukemia), DDR (DNA, damage response), KO (knockout), KD (knockdown), NA (not available), TCGA (The Cancer Genome Atlas), TSG (tumor suppressor gene). See text for details.

### 5.1 Acute myeloid leukemia, YTHDC1 condensates and mRNA stability

**FIGURE 6:**
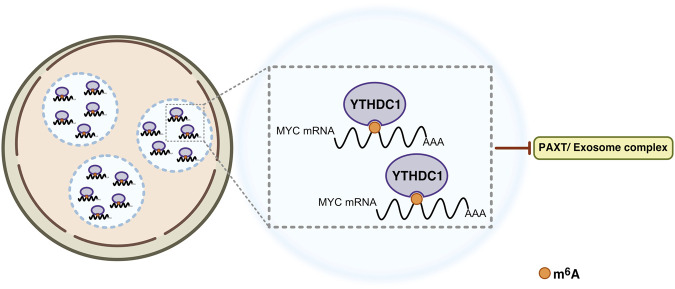
YTHDC1 forms nuclear condensates that facilitates stabilization of oncogenic mRNAs in leukemia. In the nucleus of AML cells, YTHDC1 recognizes m^6^A-modified oncogenic transcripts, such as *MYC* mRNA and promotes the formation of nuclear condensates through liqui-liquid phase separation. These m^6^A-YTHDC1-driven nuclear condensates protect specific oncogenic mRNAs from degradation by the PAXT or NEXT complexes, thereby revealing a novel oncogenic function for m^6^A-YTHDC1 condensates (see text for details).

Cheng *et al* identified a crucial role for YTHDC1 in stabilization of oncogene transcripts, such as *MYC*, within nuclear condensates that they have called nYACs (for nuclear YTHDC1-associated condensates). Starting with a genome-wide CRISPR screen in acute myeloid leukemia (AML) cell lines and expression analysis in patient samples, a critical role for YTHDC1 in oncogenic signaling was identified in AML (Cheng et al., 2021). A remarkable finding was that YTHDC1, through its IDR domains, guides the formation of nuclear condensates termed nYACs. The number of nYACs was found to be increased in AML cells compared with normal hematopoietic stem and progenitor cells. Furthermore, AML cells required nYACs to maintain cell survival and the undifferentiated state that is critical for leukemia maintenance. Mechanistically, nYACs functioned to stabilize key mRNA transcripts, including MYC, in an m^6^A-dependent manner by promoting protection from degradation by PAXT (PolyA tail Exosome Targeting) or NEXT (Nuclear Exosome Targeting) RNA degradation complexes (Cheng et al., 2021) (Figure 6).

By using a different strategy, Sheng *et al* have also uncovered a role for m6A-YTHDC1 in AML. Briefly, the authors performed reanalysis of public datasets (GEPIA2 and BloodSpot) in patients with AML, combined with AML cell line analysis, to investigate *YTHDC1* expression compared to healthy controls. Moreover, YTHDC1 depletion experiments in AML cell lines (NB-4, Kasumi-1 and MOLM-13) resulted in reduced cell growth and promoted apoptosis. Xenograft experiments in mice using *YTHDC1* WT, *YTHDC1* heterozygous knockout (HET), and *YTHDC1* KO cell lines demonstrated that YTHDC1 is required for leukemogenesis, leading to peripheral blood, liver and spleen infiltration. A transcriptomic analysis comparing MOLM-13 cell lines expressing sh*YTHDC1* to controls coupled with MeRIP-qPCR and *YTHDC1*-RIP-qPCR confirmed *MCM2*, *MCM4*, *MCM5* (Minichromosome maintenance component), and *CHAF1A* (Chromatin Assembly Factor 1 subunit A) as m^6^A-modified and bound by YTHDC1. YTHDC1 haploinsufficiency led to G1/S transition blockade, which could be rescued by the re-expression of MCM4. Finally, YTHDC1 was identified as a regulator of *MCM* subunit expression in an m^6^A-dependent manner (transcript stabilization), thus favoring maintenance of the leukemic stem cell pool through MCM-complex-mediated cell cycle regulation. This study thus echoes findings from the Kharas laboratory in that transcript-stabilizing functions of YTHDC1 are crucial to leukemia maintenance and proliferation (Sheng et al., 2021).

### 5.2 Acute myeloid leukemia, YTHDC1 and polycomb silencing by PRC2

As discussed above, m^6^A marks can be deposited on carRNAs by METTL3 or other methylases and this regulates chromatin state and transcription. Insight has recently been obtained as to how METTL3 is recruited to distinct chromatin loci. Indeed, by an extensive epigenomic data mining approach, RBFOX2, a well-studied RNA-binding protein, was identified as a chromatin factor that preferentially recognizes m^6^A on carRNAs (Dou et al., 2023). Specifically, RBFOX2 can recruit RBM15 to facilitate methylation of paRNAs. Importantly, RBM15 was also demonstrated to physically interact with YTHDC1 and to recruit polycomb repressive complex 2 (PRC2) to the RBFOX2-bound loci for chromatin silencing and transcription suppression. Furthermore, this RBFOX2/m^6^A/RBM15/YTHDC1/PRC2 axis plays a critical role in myeloid leukemia (K562 cell model of chronic myeloid leukemia). Quite strikingly, downregulation of RBFOX2 inhibits survival/proliferation of acute myeloid leukemia cells and promotes their myeloid differentiation. RBFOX2 is also required for self-renewal of leukemia stem/initiation cells and AML maintenance (Dou et al., 2023). RBFOX2 overexpression is associated to poor survival. Taken together, the RBFOX2/m^6^A/RBM15/YTHDC1/PRC2 pathway is identified as critical oncogenic signaling hub in AML with potential therapeutic implications.

### 5.3 YTHDC1 and B-ALL

B-cell acute lymphoblastic leukemia (B-ALL) is an aggressive malignancy characterized by the aberrant accumulation of immature and dysfunctional B cells in bone marrow (BM). Treatment resistance is problematic (Li et al., 2025). In this setting, *YTHDC1* expression was screened in B-ALL and found to be highly expressed. Functional assays showed that YTHDC1 knockdown significantly increased the accumulation of endogenous and chemotherapeutic agent-induced DNA damage in B-ALL cells. YTHDC1 was shown to bind to and stabilize m^6^A-modified *KMT2C* mRNA which encodes KMT2C, a key enzyme catalyzing histone H3K4 methylation required for the expression of DNA damage response (DDR)-related genes. Reasoning that YTHDC1 inhibitors might improve chemotherapy by attenuating DDR via reducing KMT2C, molecular docking and biochemical experiments were undertaken and identified EPZ-5676 as a YTHDC1 inhibitor. This is somewhat unexpected since EPZ-5676 is defined as an inhibitor of the histone H3K79 methyltransferase, DOT1L. Nonetheless, combination of EPZ-5676 with Cytarabine (Ara-c) significantly improved the efficacy of chemotherapy in B-ALL mouse models in YTHDC1 ‘high’ B-ALL cells. Collectively, this work reveals that YTHDC1 is required for DDR in B-ALL cells by upregulating DDR-related gene expression via stabilization of m^6^A-modified *KMT2C* mRNA, and consequent increased histone H3K4 methylation. Targeted inhibition of YTHDC1 is thus a potentially new therapeutic strategy against B-ALL and possibly other cancers as well (Li et al., 2025).

Taken together, these studies have derived some major overarching principles regarding cell intrinsic YTHDC1 function in leukemia, identifying it as a stabilizer of oncogenic transcripts, a recruiter of chromatin modifiers, and a component of nuclear condensates. It remains to be seen whether these functions operate in other blood cancers, notably lymphoma. Whether YTHDC1 might also exert tumor suppressor activity in certain blood cancers will also need to be addressed as will its impact in the leukemia microenvironment.

## 6 Conclusion

In addition to its role in the control of RNA stability, splicing and nuclear export, m^6^A-YTHDC1 is emerging as playing multifaceted roles in a complex array of RNA-chromatin cross-talks that regulate chromatin state, topology and multiple genome functions, including gene silencing and activation, and genome stability. A unique aspect of m^6^A-YTHDC1 function is its ability to organize nuclear condensates which, at least in theory, and according to their molecular composition, could dynamically drive multiple facets of m^6^A-YTHDC1 activity, simultaneously, in the same cell nucleus. A crucial question will be how cell context, developmental or differentiation stage specific cues might fine tune these diverse regulatory activities to achieve desired outputs in normal and disease states, particularly cancer, where epitranscriptomic malfunction, including m^6^A-YTHDC1, is emerging as a hallmark of cancer. Specifically regarding m^6^A-YTHDC1, it will be important to examine whether its exclusively nuclear localization can be altered (e.g., under specific stress, in certain cancers). Thus far this has not been investigated. Key unanswered questions remain. For example: what determines whether YTHDC1 acts as a transcriptional activator or repressor in a given context? How are its m^6^A-dependent and independent functions integrated? What is the therapeutic potential and challenge of targeting a multifaceted protein like YTHDC1, given its essential roles in normal cells? How best could YTHDC1 inhibitors and other epitranscriptome active agents be tested in the clinical setting? These questions and more are already under intense discussion in the field. New basic and clinical research efforts are needed to bring much-needed answers.
